# Thermoresponsive Physically Cross-Linked Hydrogels with Bidirectional Optical Response for Smart Windows Application

**DOI:** 10.1021/acsami.5c14919

**Published:** 2025-09-17

**Authors:** Zeyu Zhang, Aifang Yao, Zao Cheng, Patrizio Raffa

**Affiliations:** † Smart and Sustainable Polymeric Products, Engineering and Technology Institute Groningen (ENTEG), 3647 Faculty of Science and Engineering; University of Groningen, Nijenborgh 3, 9747 AG Groningen, The Netherlands; ‡ College of Biological Science and Engineering, 12423 Fuzhou University, Fuzhou 350108, China

**Keywords:** hydrogels, thermoresponsive, smart window, self-healing, antifreezing

## Abstract

Hydrogel-based smart windows have the potential to reduce the energy consumption associated with air conditioning and lighting systems. Most existing hydrogels exhibit a unidirectional response to a specific temperature, limiting their applicability. In this work, a bidirectional temperature-responsive hydrogel was developed by incorporating hydroxypropyl cellulose (HPC) into a physically cross-linked copolymer matrix of *N*-(2-hydroxyethyl) acrylamide (HEAA) and acrylamide (AM), with cetyltrimethylammonium bromide (CTAB) used to stabilize lauryl methacrylate (LMA) micelles in a deep eutectic solvent (DES)/H_2_O binary solvent system. At higher temperatures, the hydrogel becomes opaque through phase separation driven by the lower critical solution temperature (LCST) of HPC. At lower temperatures, another optical transition seems to be governed by the growth of micelles formed from LMA. The temperature operating window can be tuned by changing the composition, keeping a rapid optical switching response of less than 30 s. Furthermore, due to the dynamic reversibility of hydrophobic associations and hydrogen bonding, the hydrogel exhibits excellent mechanical strength and self-healing capability at room temperature. The presence of the DES also contributes to its antifreezing performance, allowing the hydrogel to retain flexibility even at −20 °C. With its integrated functionalities, this material represents a highly promising candidate for smart window applications in real-world environments.

## Graphical abstract

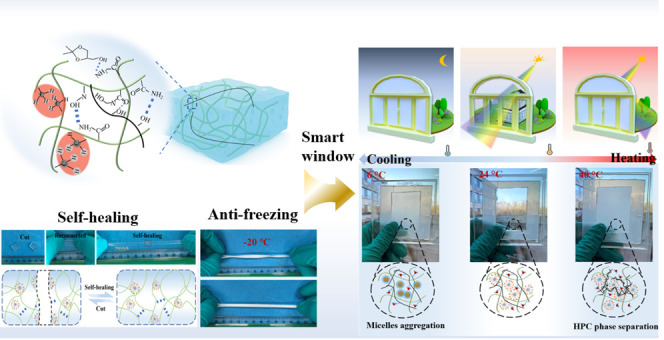

## Introduction

Smart polymeric hydrogels have garnered significant attention due to their exceptional responsiveness to external stimuli, including voltage, light intensity, and temperature.^1–3^ Among these, temperature responsiveness stands out as it requires no additional substances, offers ease of control, and remains cost-effective.^4,5^ Temperature-responsive phase separation is a common phenomenon in hydrogel systems and is typically classified into two categories: lower and upper critical solution temperatures (LCST and UCST, respectively). As the temperature changes, the polymer chains undergo a transition between hydrophilic and hydrophobic states, resulting in phase separation.^4,6^ A widely utilized polymer for thermochromic smart hydrogels is poly­(*N*-isopropylacrylamide) (PNIPAM) owing to its near-room-temperature LCST of 32 °C, rapid phase transition kinetics, and excellent solar modulation properties.^7–9^ However, conventional chemically cross-linked PNIPAM-based materials typically exhibit higher phase separation temperatures and often require multistep fabrication processes, rendering their preparation complex and resource-intensive.^10^ Hydroxypropyl cellulose (HPC), an interesting cellulose derivative, demonstrates excellent low-temperature hydrophilicity and biocompatibility, making it another effective phase change material.^11–13^ When the temperature exceeds the LCST (45 °C), HPC transitions into a globular form and precipitates from the solvent due to hydrophobic interactions within its polymer chains, endowing it with distinctive temperature-responsive capabilities.^14,15^ However, poor thermal stability and a slow phase transition rate significantly hinder the further development of smart window applications. Although some HPC-based smart hydrogels have been reported recently, their chemically cross-linked networks often result in a relatively high phase transition temperature^16^ or lead to undesirable shrinkage after the transition.^17^

In contrast, the chromic mechanism of thermochromic hydrogels derived from micelles, like sodium dodecyl sulfate (SDS), follows an opposite trend, where transparency increases with rising temperature. In the precursor solution of the hydrogel, SDS forms micelles that function as thermoresponsive units.^18^ These micelles become physically confined within the three-dimensional cross-linked network of the hydrogel. The surrounding hydrogel matrix, which possesses high optical clarity, maintains its transparency while embedding responsive micellar structures, enabling the hydrogel to exhibit a temperature-dependent optical behavior. At low temperatures, SDS micelles spontaneously aggregate within the hydrogel matrix, resulting in light scattering and reduced transparency, thereby rendering the hydrogel opaque. By adjusting the micelle formation conditions, the thermochromic transition temperature (*T*_c_) could be precisely controlled. Inspired by this mechanism, other surfactants such as sodium lauryl sulfate (SLS), sodium dodecylbenzenesulfonate (SDBS), and hexadecylpyridinium bromide (HPB) have also been explored for their thermochromic behavior.^19–21^ However, to the best of our knowledge, thermoresponsive hydrogels utilizing cetyltrimethylammonium bromide (CTAB) micelles for smart window applications have not yet been reported.

Due to their inherent softness, hydrogels used in smart windows are susceptible to mechanical damage during prolonged use. This can compromise their structural integrity, causing a decline or even complete loss of mechanical properties and ultimately shortening their service lifespan.^22–24^ The integration of a self-healing function allows damaged hydrogels to autonomously repair, restoring their original mechanical properties and conductivity, thus prolonging their service life. Supramolecular networks driven by noncovalent interactions, such as hydrogen bonding, electrostatic interactions, and hydrophobic associations, endow hydrogels with self-healing capabilities and superior mechanical performance.^25^ The physical and chemical properties of such physical cross-links are highly tunable by adjusting the composition, structure, and flexibility of the monomers. This approach can be achieved with ease, avoiding the need for complex procedures and harsh reaction conditions. However, research on smart hydrogel windows formed solely through physical cross-linking remains limited. Moreover, in practical applications, the high water content of hydrogels makes them susceptible to freezing at subzero temperatures, resulting in a loss of smart functionality and the formation of cracks in the windows, which pose safety risks to end users.

Another issue is that most current smart hydrogel windows exhibit limited responsiveness to a single temperature threshold. This limitation renders them unsuitable for dual functionality, namely, energy savings during the day and privacy protection at night.^26,27^ Bidirectional temperature response refers to materials that maintain high transparency within a specific temperature range. As the temperature either increases or decreases, these materials progressively reduce transparency, aligning more closely with practical application needs.^28^ Some studies have successfully developed hydrogels with bidirectional temperature response properties that meet these dual requirements. However, these materials often involve complex multistep processes^29^ or require the introduction of salts or chemical cross-linkers, which can disrupt hydrogen bonding between polymer chains and water molecules and negatively affect visible light transmittance.^30,31^ Therefore, developing a simple method to prepare a hydrogel only with a physically cross-linked network that responds to bidirectional temperature changes and multifunctionality remains a significant challenge, particularly for smart window applications, where such functionality is crucial.

Herein, by embedding HPC into a physically cross-linked *N*-(2-hydroxyethyl) acrylamide (HEAA)–AM copolymer and using CTAB to stabilize lauryl methacrylate (LMA) micelles within a deep eutectic solvent (DES)/H_2_O binary solvent, we fabricated a hydrogel that responds to temperature changes in two directions. This hydrogel exhibits energy-efficient performance at elevated temperatures, with phase separation induced by the disruption of hydrogen bonding between HPC and water upon heating. By incorporating CTAB micelles into a cross-linked copolymer composed of hydrophilic poly­(HEAA-*co*-AM) (PHA) and hydrophobic LMA, micelle aggregation is promoted at low temperature, leading to an increase in micelle size and a significant enhancement in light-blocking performance. This results in an opaque state at low temperatures, effectively ensuring user privacy. The hydrogel’s dual response temperatures can be independently controlled over a broad temperature range, with the LCST adjustable from 24.8 to 39.6 °C by precisely tuning the LMA concentration. Furthermore, owing to the dynamic reversibility of hydrophobic interactions and hydrogen bonding, the hydrogels exhibit self-healing capabilities at room temperature, enabling the restoration of their original transparency and solar modulation performance. At low temperatures, conventional hydrogels tend to freeze, making them unsuitable for smart window applications in buildings. The incorporation of DES effectively addresses this issue as solketal in DES forms hydrogen bonds with water molecules, preventing the formation of ice crystals and ensuring the hydrogel’s functionality under subzero conditions. Benefiting from strong interfacial interactions with substrates, the hydrogels demonstrate exceptional adhesion, facilitating the efficient manufacturing and packaging of smart windows.^32^ Given its dual functionality in energy conservation and privacy protection, the novel bidirectional temperature-responsive hydrogel emerges as a highly promising candidate for smart windows, offering substantial potential for real-world applications.

## Results and Discussion

### Fabrication of Thermochromic Hydrogels

In the DES/H_2_O binary solvent, CTAB stabilized LMA to form micelles, followed by the addition of HPC, HEAA, and AM to achieve a homogeneous solution. Subsequent photopolymerization yielded a pure physically cross-linked transparent hydrogel. As illustrated in Figure 1, when the ambient temperature was between *T*_c_ and LCST, the thermoresponsive hydrogel was transparent, allowing light to pass through. When the temperature was lower than *T*_c_ (at 6 °C), CTAB micelles in the hydrogel’s three-dimensional network spontaneously aggregated and formed micellar clusters, causing the smart window to transition into an opaque state. Above the LCST, hydrogen bonds between HPC and water began to weaken, leading to intensified light scattering. Figure S1 presents photographs of smart windows based on covalently cross-linked (cross-linked by MBA) and noncovalently cross-linked hydrogels, both in situ polymerized in a DES/H_2_O binary solvent system. The covalently cross-linked hydrogel window remained transparent at 30 °C and only became opaque upon heating to 35 °C, while the noncovalently cross-linked hydrogel exhibited an opaque state already at 30 °C, indicating a lower phase transition temperature. The hydrogel was designated as PHAL_*X*_/DES_*Y*_/HPC_*Z*_, where *X*, *Y*, and *Z* represent the mass percentages of LMA, DES, and HPC relative to that of water, respectively. For example, PHAL_5_/DES_20_/HPC_4_ corresponds to a hydrogel formulation containing 5% LMA, 20% DES, and 4% HPC. Unless otherwise specified, a 1:1 molar ratio of HEAA to AM was used in the subsequent experiments.

**Figure 1:**
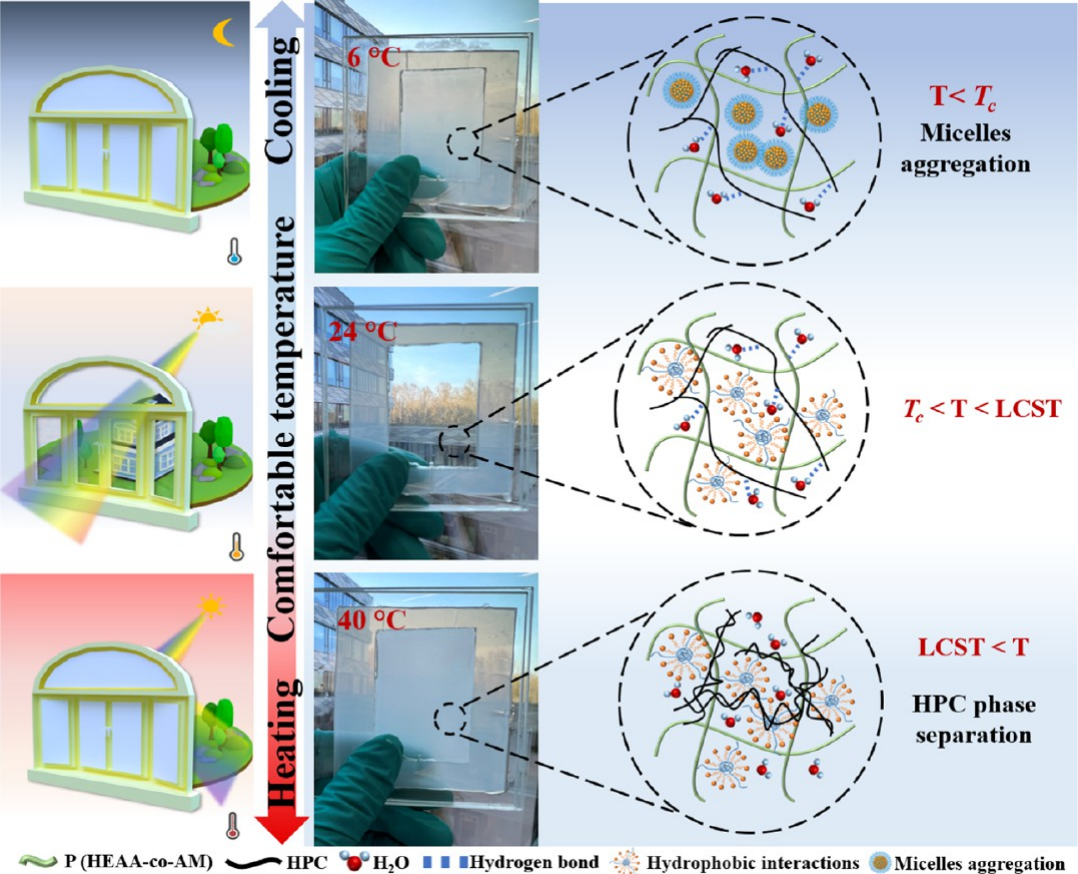
Schematic illustrations and corresponding photos of the hydrogel at different temperatures.

The internal microstructure of the hydrogel is presented in Figure 2a. At low temperatures, micelle aggregation induces the formation of a densely cross-linked network, rendering the hydrogel opaque, which effectively ensures privacy protection. At 24 °C, the network exhibits large and interconnected pores that allow incident light to pass through, resulting in a transparent state. Upon heating to 40 °C, the hydrogel underwent a high-temperature phase transition, leading to a more compact network structure and a corresponding loss of transparency. Energy-dispersive X-ray spectroscopy (EDS) elemental mapping confirms the homogeneous dispersion of the DES within the polymer matrix, with C, O, N, and Cl elements uniformly distributed throughout the hydrogel (Figure 2b), which was beneficial to forming the homogeneous and dense dynamic sacrificial bonds. More details of the chemical composition of the hydrogel were investigated by X-ray photoelectron spectroscopy (XPS). As shown in Figure 2c, the existence of Cl elements was observed in the hydrogel after the introduction of DES. From the high-resolution N 1s spectrum (Figure 2d), a new peak at 402.2 eV is observed in PHAL_5_/DES_20_/HPC_4_, which is absent in PHAL_5_/HPC_4_, and can be attributed to the N^+^ originating from the DES component, confirming the successful incorporation of DES. Figure 2e presents the high-resolution spectrum of C 1s. The PHAL_5_DES_20_ hydrogel had four characteristic peaks at 288.9, 286.2, 285.3, and 284.8 eV, attributed to C=O, C–OH, C–N, and C–C, respectively. In the PHAL_5_/DES_20_/HPC_4_ hydrogel, a decrease of the C=O binding energy, along with increases in the C–O and C–N binding energies, indicates the formation of hydrogen bonds between HPC and the PHA polymer chains.^33,34^ This is further supported by the O 1s spectrum, where a shift to lower C=O binding energy provides additional evidence for hydrogen bonding within the hydrogel network (Figure 2f).^35^ The internal molecular interactions were investigated by Fourier transform infrared (FTIR) spectroscopy (Figure 2g). The –OH stretching peak of the PHAL_5_ hydrogel, originally located at 3323 cm^–1^, shifted to 3346 cm^–1^ following the introduction of DES. This blue shift suggests the formation of hydrogen bonds between hydroxyl groups and Cl^–^ ions in the DES of the PHAL_5_/DES_20_ hydrogel. Additionally, the observed weakening of the C=O stretching vibration at 1635 cm^–1^ may result from hydrogen bonding between the hydroxyl groups of HPC and the amide groups in the PHA polymer chains with the introduction of HPC into the hydrogel network. Notably, no new characteristic peaks are observed in the composite hydrogel, indicating that no new covalent bonds are formed between PHA and HPC.^26^

**Figure 2:**
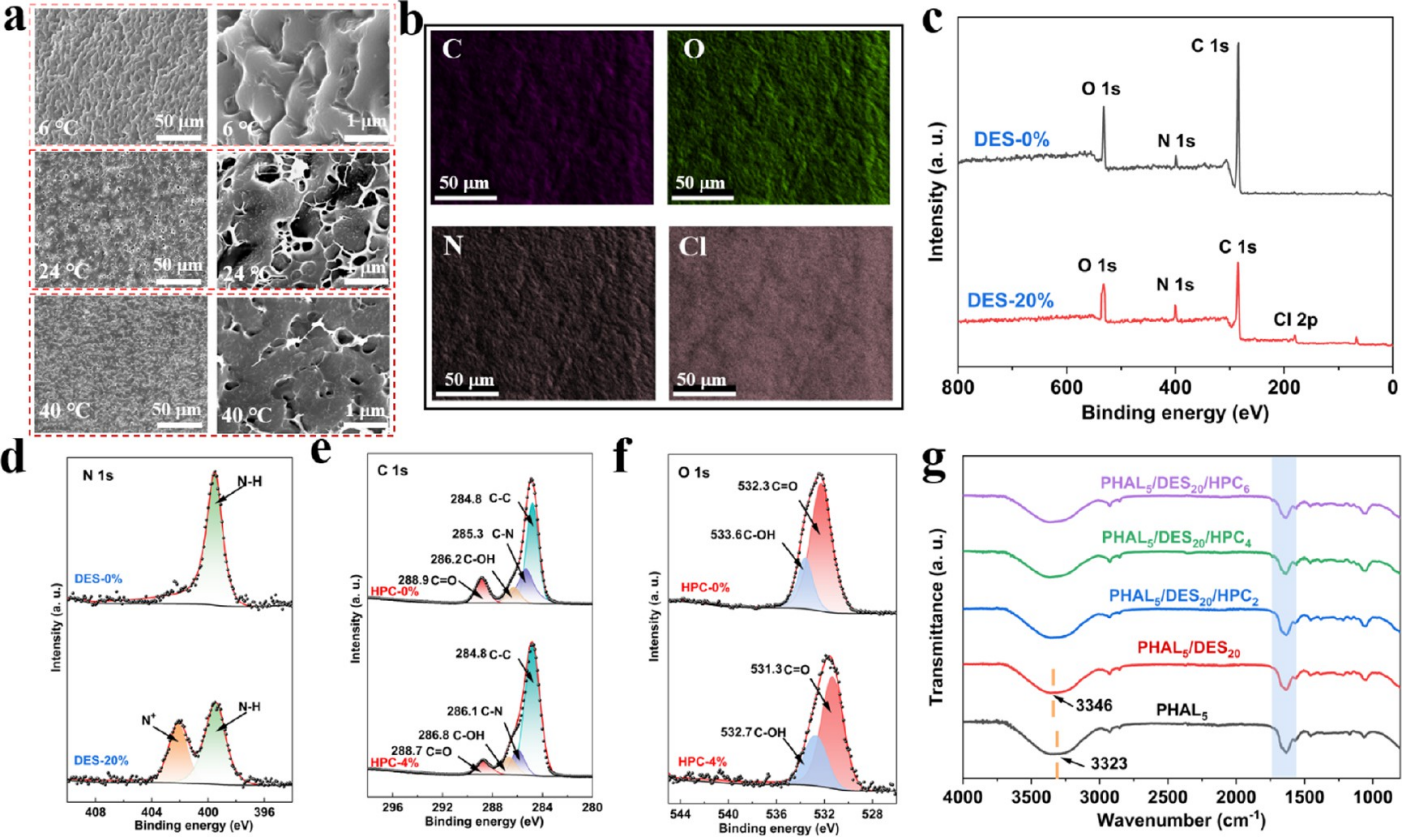
(a) SEM images of the PHAL_5_/DES_20_/HPC_4_ hydrogel at 6, 24, and 40 °C. (b) Elemental maps of PHAL_5_/DES_20_/HPC_4_ hydrogels. (c) XPS survey of the PHAL_5_/HPC_4_ hydrogel and PHAL_5_/DES_20_/HPC_4_ hydrogel. (d) N 1s spectra of the PHAL_5_/HPC_4_ hydrogel and PHAL_5_/DES_20_/HPC_4_ hydrogel. (e) C 1s XPS spectra of the PHAL_5_/DES_20_ hydrogel and PHAL_5_/DES_20_/HPC_4_ hydrogel. (f) O 1s XPS spectra of the PHAL_5_/DES_20_ hydrogel and PHAL_5_/DES_20_/HPC_4_ hydrogel. (g) FTIR spectra of PHAL_5_, PHAL_5_/DES_20_, and PHAL_5_/DES_20_/HPC_Z_ hydrogels.

### Thermochromic Mechanism

Photographs of the control and composite hydrogels containing varying concentrations of HPC at different temperatures are presented in Figure 3a. In the absence of HPC, the hydrogel retained transparency even at 40 °C. In contrast, the incorporation of HPC (4%) endowed the hydrogel with distinct thermoresponsive behavior; it was uniformly transparent at 24 °C but turned opaque at 40 °C. The internal structure of the hydrogel reveals that HPC contains thermoresponsive hydrophilic and hydrophobic groups, with the ability of the hydrophilic groups to form hydrogen bonds with water being temperature-dependent. When the temperature exceeds the LCST, hydrogen bonds between HPC and water begin to weaken, promoting the aggregation of hydrophobic groups. This transition induces a macroscopic phase separation, resulting in the hydrogel becoming opaque.^37^

**Figure 3:**
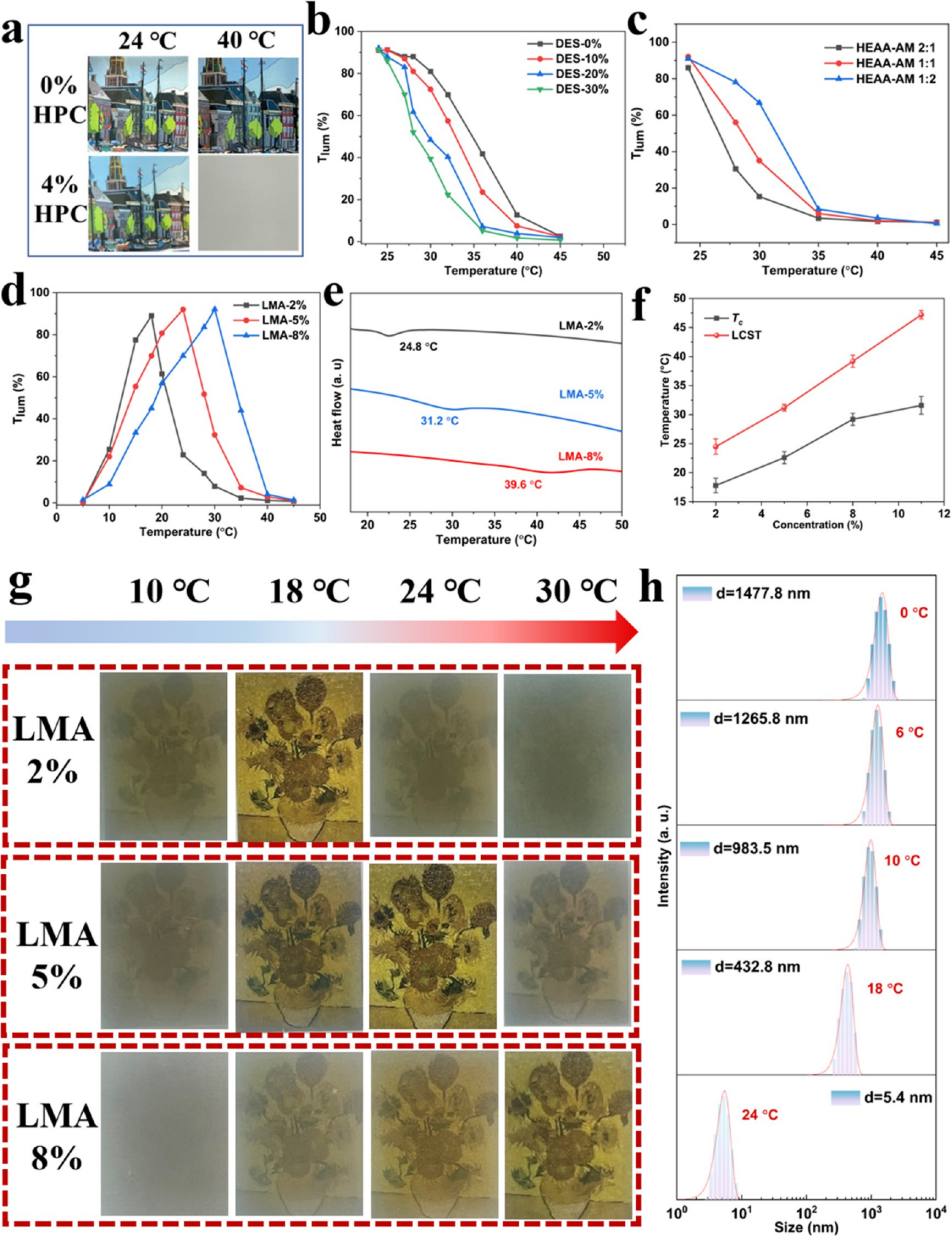
(a) Photos of PHAL_5_/DES_20_ and PHAL_5_/DES_20_/HPC_4_ at 20 and 40 °C. The influence of (b) DES content, (c) molar ratio of HEAA and AM, and (d) LMA concentration on the *T*_lum_ of the hydrogel. (e) DSC curves of PHAL_*X*_/DES_20_/HPC_4_ hydrogels with different LMA concentrations. (f) *T*_c_ and LCST of PHAL_*X*_/DES_20_/HPC_4_ with different LMA concentrations. (g) Optical images of PHAL_*X*_/DES_20_/HPC_4_ hydrogels with different LMA concentrations at different temperatures. (h) The particle size distribution of PHAL_5_/DES_20_/HPC_4_ at different temperatures.

In addition, the optical transmittance of the hydrogel at a given temperature can be tuned by adjusting the content of DES, as shown in Figure 3b. Specifically, increasing the DES concentration results in a lower integral luminous transmittance *T*_lum_ (380–780 nm) at the same temperature. This occurred because HPC chains partially engage in hydrogen bonding interactions with the DES, disrupting existing hydrogen bonds between HPC chains and water molecules. At higher DES concentrations, the weakened hydrogen bonding between HPC and water molecules promotes stronger polymer and HPC interactions, consequently reducing the lower transparency of the hydrogel and accelerating the HPC’s phase transition. The variations in the hydrogel’s light transmission interval as a function of the molar ratio of HEAA to AM are shown in Figure 3c. As the molar ratio of HEAA to AM changes from 2:1 to 1:2, the temperature at which the hydrogel became opaque gradually increased. With higher AM content, the hydrophilicity of the hydrogel network is enhanced,^16^ allowing more water molecules to interact strongly with the polymer chains. This increased level of hydration stabilizes the hydrated state of HPC and suppresses its hydrophobic aggregation. Consequently, a higher temperature is required to disrupt these interactions and induce phase separation.

For smart window applications, a wider operating temperature range is desirable to accommodate the varying climatic conditions of the different regions. Benefiting from the noncovalent cross-linked in the hydrogel network without the addition of external cross-linkers, the LCST and *T*_*c*_ can be effectively tuned by altering the LMA content. As illustrated in Figure 3d, the temperature interval for optical transmission broadens when the LMA concentration increases from 2 to 8%. According to differential scanning calorimetry (DSC) measurements (Figure 3e), the LCST continuously shifted from 24.8 to 39.6 °C, demonstrating precise control over the hydrogel’s LCST. The impact on the micelles state transition of *T*_c_ is from 17.8 to 30.6 °C (Figure 3f), meeting the climatic adaptability requirements of diverse regions and enabling versatile tunability for practical applications. Photographs illustrating the optical appearance of hydrogels with varying LMA contents at temperatures of 10, 18, 24, and 30 °C are presented in Figure 3g. At 10 °C, all hydrogel samples appeared opaque, significantly obscuring the visibility of the flower placed beneath. The hydrogel containing 2 wt % LMA transitioned from opaque to transparent at 18 °C and then exhibited a clear switch back to an opaque state at 24 °C. In comparison, increasing the LMA content to 5 wt % delayed this optical transition, with the hydrogel becoming transparent at 24 °C and turning opaque upon further heating to 30 °C. Increasing LMA concentrations promoted the formation of a greater number of micelles, thus strengthening hydrophobic interactions among polymer chains and forming a more stable hydrogel network. Consequently, this resulted in an upward shift in the transition temperature, turning the hydrogel opaque at relatively higher temperatures.

The temperature responsiveness of the hydrogel at low temperatures is governed by the aggregation behavior of micelles formed from LMA stabilized by CTAB. As the temperature decreases, micelle size increases, enhancing the light-blocking properties. Dynamic light scattering (DLS) measurements were conducted to further elucidate this mechanism. Figure 3h reveals that upon cooling below 10 °C, micelle sizes increased significantly to over 800 nm, larger than the wavelength of visible light (380–780 nm), resulting in strong scattering of incident light and causing the hydrogel to become opaque at low temperatures. In contrast, when warmed above the critical temperature, micelle size reduced markedly, enabling the hydrogel to revert to transparency. The precursor solution of the hydrogel containing CTAB-stabilized LMA micelles became opaque when cooling to 6 °C, whereas the solution without CTAB micelles or with the addition of the chemical cross-linker MBA remained transparent. This difference in optical behavior persisted after polymerization (Figure S2). These findings highlight the intricate interplay between hydrophobic associations and micelle formation, demonstrating the potential to achieve precise control of the optical performance for smart window applications. In this system, modulation of *T*_c_ and LCST is intrinsically coupled due to the noncovalent cross-linking of CTAB-assisted LMA micelles. Increasing LMA content strengthens hydrophobic interactions, yielding a denser network and shifting the LCST upward while also facilitating micelle aggregation that affected *T*_c_. The independent regulation of *T*_c_ and LCST would significantly broaden the potential applications of hydrogels, and we will continue to improve this aspect of our work.

### Bidirectional Temperature Responsiveness

To evaluate the bidirectional optical properties of the hydrogel, its transmittance in the visible spectrum was measured at various temperatures. As shown in Figure 4a, the hydrogel exhibited high transparency, maintaining transmittance above 80% across the entire visible range at temperatures between 20 and 26 °C. This performance satisfies the standard requirement for architectural glass, which stipulates that light transmittance should not fall below 60%. The hydrogel showed the highest *T*_lum_ = 92% at 24 °C (Figure 4b). In contrast, when temperatures were below 15 °C, it became opaque, with *T*_lum_ dropping below 60%. A similar decline in *T*_lum_ was observed at elevated temperatures (>28 °C), confirming the hydrogel’s bidirectional temperature responsiveness. At a comfortable temperature range of 18–26 °C, the hydrogel maintains high transmittance, ensuring an unobstructed view, which is essential for smart window applications. Figure S3 demonstrates that the hydrogel exhibited a bidirectional optical response over a broad temperature range. The UV–vis–NIR transmittance spectra were employed to evaluate the optical properties of the hydrogel across a wavelength range of 280–2500 nm, with the sample thickness fixed at 2 mm (Figure 4c,d). The two decreases in the transmittance at 1430 and 1930 nm, corresponding to the O–H stretching in water and the binding of O–H stretching to the H–O–H bending. At 24 °C, the solar transmittance (*T*_sol_) of the hydrogel was 91.4%, indicating excellent transparency under ambient conditions. At 6 °C, enhanced hydrophobic interactions drive the aggregation of LMA domains within CTAB micelles, leading to an increase in micelle size and enhanced light scattering, which reduces transmittance (*T*_sol_ = 7.8%). As the temperature increases, the hydrogel gradually becomes opaque; at 40 °C, *T*_sol_ decreases significantly to 9.8%. The resulting Δ*T*_sol_ reaches as high as 81.6% (Δ*T*_LCST_) and 82.9% (Δ*T*_c_), respectively, effectively reducing the solar heat gain. Notably, the *T*_lum_ and Δ*T*_sol_ of the hydrogels surpass those of most previously reported thermotropic hydrogel-based smart windows (Figure 4e),^5,9,16,29,30,32,38–41^ highlighting the superior optical performance of this system.

**Figure 4:**
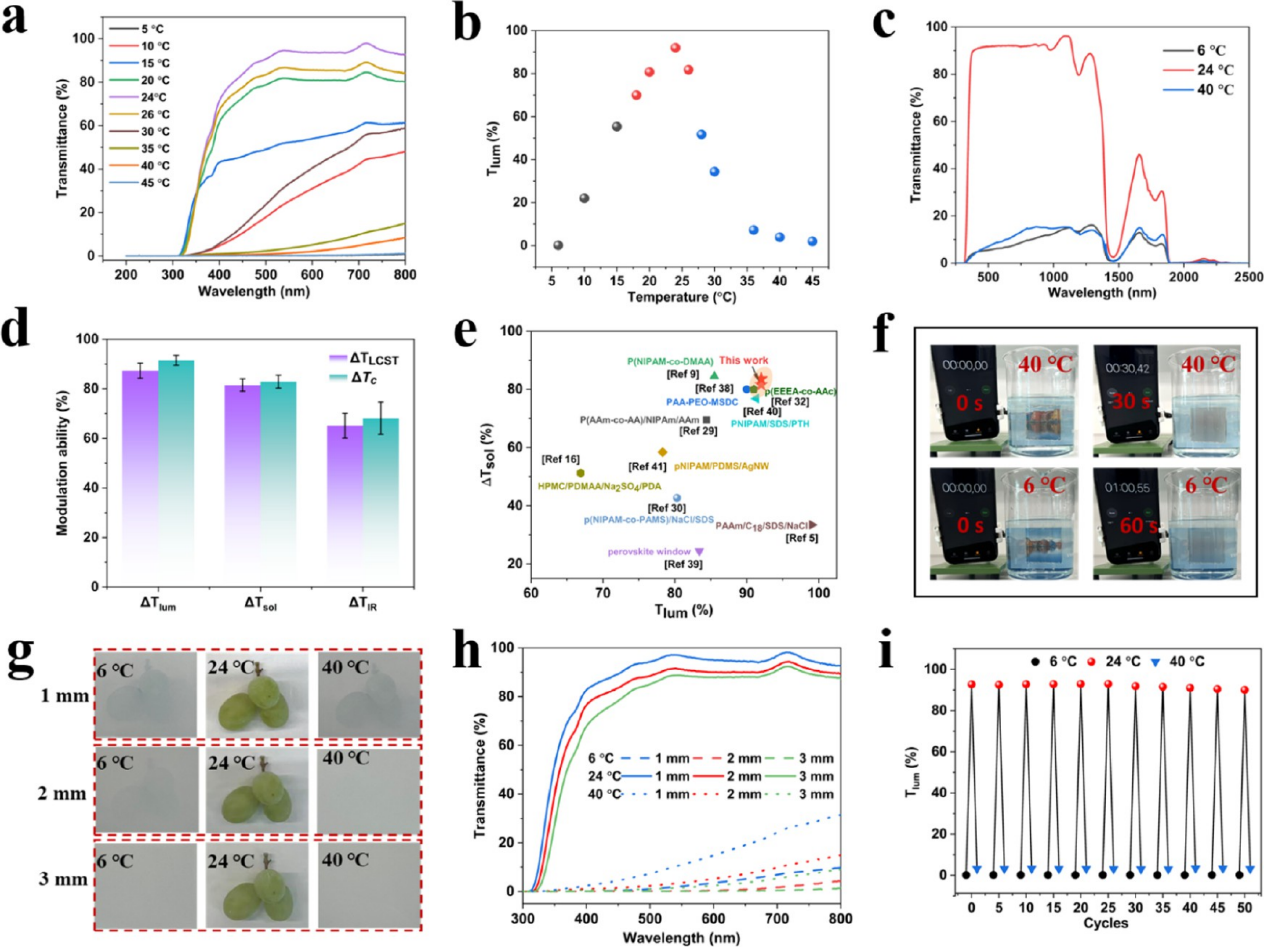
(a) Transmittance spectra of the hydrogel at different temperatures. (b) *T*_lum_ of the hydrogel at different temperatures. (c) UV–vis–NIR transmittance spectra of the hydrogel smart window. (d) Hydrogels at different phase transitions corresponding to the modulation of the light transmittance (Δ*T*_lum_, (380–780 nm), Δ*T*_IR_, (780–2500 nm), and Δ*T*_sol_, (280–2500 nm)). (e) Comparison of the transmittance and solar modulation ability of thermochromic hydrogel smart windows in recent years. (f) Response time of the hydrogel. (g) Photograph of hydrogels with various thicknesses at different temperatures. (h) Transmittance spectra of hydrogels with various thicknesses at different temperatures. (i) Thermal cycles.

In addition, the hydrogel showed a rapid thermal response. As illustrated in Figure 4f, at 6 °C, the transmittance exhibited a rapid change and stabilized within just 60 s, while at 40 °C, stabilization occurred within 30 s. Movie S1 captures the discoloration process of the hydrogel in a 6 °C water bath, while Movie S2 records the change in light transmittance when the hydrogel was exposed to 40 °C and quickly returned to being transparent at 20 °C, further demonstrating its bidirectional temperature responsiveness.

Hydrogels with varying thicknesses were prepared to investigate the influence of the thickness on optical transparency. Photographs of samples with different thicknesses at 6, 24, and 40 °C are shown in Figure 4g. At 24 °C, all samples, regardless of thickness, appear transparent, allowing clear visibility of the object behind them and indicating consistently high transmittance. At 6 and 40 °C, the degree of blurring observed for the object behind the hydrogel increases with the thickness. The detailed transmittance spectra of hydrogel samples with different thicknesses in both transparent and opaque states across the 300–800 nm wavelength range are presented in Figure 4h. At 550 nm in the transparent state, the transmittance values for the 1 mm, 2 mm, and 3 mm thick samples were 96.2%, 90.8%, and 88.3%, respectively, demonstrating high optical clarity across all thicknesses. All samples exhibited excellent bidirectional temperature responsiveness under both low and high ambient conditions. At 6 °C, the transmittance of the 1 mm sample dropped to 2.3%, while further reductions were observed with increased thickness, 1.3% for the 2 mm sample and 0.10% for the 3 mm sample. Similarly, at 40 °C, the transmittance at 550 nm was 11.0% for the 1 mm sample, decreasing to 3.7% and 1.6% for the 2 and 3 mm samples, respectively. Reversible optical transitions and long-term stability are critical performance indicators for smart window applications. As shown in Figure 4i, after 50 thermal cycles between cold and hot states, the visible light transmittance of the hydrogel remained unchanged, indicating excellent stability. The cycling test confirms that the hydrogel exhibits reliable reversibility and outstanding cyclic durability, making it a promising candidate for practical long-term use in adaptive light-regulating systems.

### Mechanical Properties

Strong mechanical properties ensure the structural integrity of the hydrogel throughout production, transportation, installation, and long-term use, enabling the development of durable and reliable thermochromic smart windows for practical applications.^27^ Even without the addition of chemical cross-linking agents, the hydrogel exhibited excellent mechanical properties. The micelles formed in the aqueous solution acted as physical cross-linking points within the hydrogel network after polymerization and, together with the dense hydrogen bonding between the polymer chains and HPC, played a key role in imparting high strength to the hydrogel (Figure 5a). Figure 5b shows that the hydrogel is able to withstand stretching and twisting. The puncture resistance test further confirms the mechanical robustness of the hydrogel. However, without LMA, the hydrogel was soft and sticky (Figure S4).

**Figure 5:**
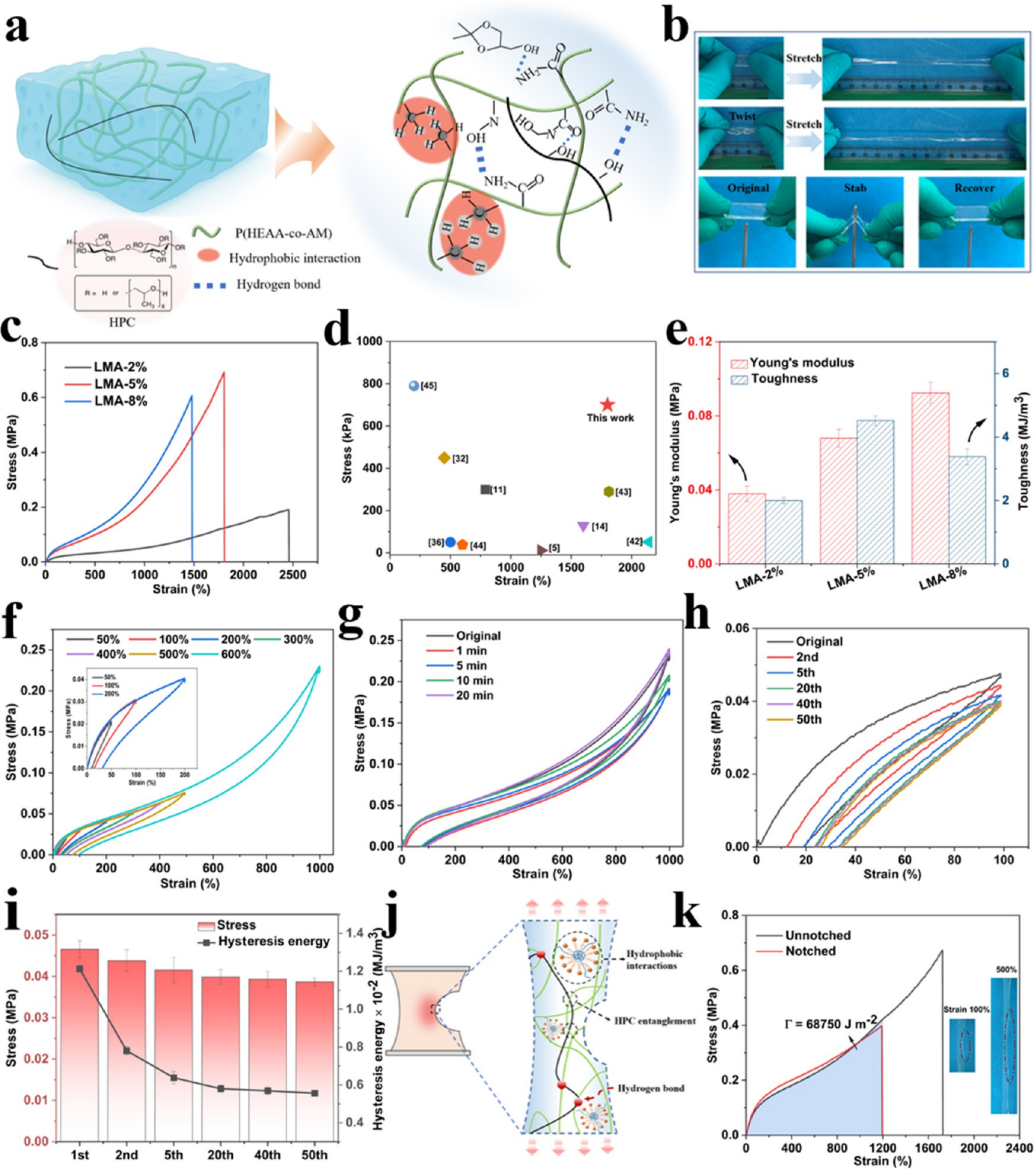
(a) Schematic illustrations of interactions in the hydrogel. (b) Photographs of the hydrogel could be stretched, twisted, and stabbed. (c) Strain curves of hydrogel with different LMA contents. (d) A comparison of stress and strain between this work and the previously reported hydrogel. (e) Young’s modulus and toughness of the hydrogel with different LMA contents. (f) Cyclic stress–strain curves of the hydrogel in various strains ranging from 50 to 1000%. (g) Stress–strain curves of the hydrogel subjected to cyclic loadings at the strain of 1000% with appropriate intervals. (h) Cyclic loading–unloading curves of the hydrogel at a tensile strain of 100%. (i) Cyclic loading–unloading stress and hysteresis energy of the hydrogel. (j) Schematic illustrations of crack resistance. (k) Tensile stress–strain curves of unnotched and notched hydrogels.

By precisely tuning the LMA content, the hydrogels exhibit a wide range of adjustable mechanical properties, with tensile strain values ranging from 1479% to 2458% and tensile stress values between 0.68 and 0.19 MPa (Figure 5c). Increasing the LMA concentration leads to a higher cross-linking density, which enhances the tensile strength. However, when the cross-linking density becomes high, as observed at LMA values of 4 and 8%, the mobility of the polymer chains is significantly restricted, resulting in a reduction in stretchability. The hydrogel demonstrates superior tensile strength and stretchability compared to previously reported thermoresponsive hydrogels, including those cross-linked with conventional chemical agents (Figure 5d).^5,11,14,32,36,42–45^ The hydrogel prepared with 2% LMA exhibited a Young’s modulus of 0.038 MPa and a toughness of 2.0 MJ m^–3^ (Figure 5e). Increasing the LMA content to 5% resulted in a higher Young’s modulus of 0.058 MPa and an enhanced toughness of 4.5 MJ m^–3^, indicating improved stiffness and flexibility. However, further increasing the LMA concentration to 8% stiffened the hydrogel, leading to reduced flexibility, as evidenced by a decrease in toughness to 3.4 MJ m^–3^. The resulting over-cross-linking severely restricts polymer chain mobility, suppressing reversible chain rearrangements and energy dissipation mechanisms during deformation, which ultimately leads to a reduction in toughness.

The loading–unloading curves at predefined strain levels exhibited minimal hysteresis, indicating efficient energy dissipation during cyclic deformation (Figure 5f). The reversible interactions effectively absorb and dissipate mechanical energy, contributing to the hydrogel’s robust mechanical performance and resilience.^46,47^ At a strain of 1000%, the hydrogel demonstrated the highest dissipated energy (Figure S5), reflecting the progressive rupture of a greater number of hydrogen bonds under larger deformations. The presence of hydrophobic associations of LMA segments served as cross-linking sites within the hydrogel network, ensuring remarkable elasticity. As a result, the hydrogel exhibited excellent recovery efficiency, reaching nearly its original state within 12 min under 500% strain and within 20 min under 1000% strain (Figures 5g and S6). Under 50 continuous cycles at a strain of 50%, the tensile strength exhibited a gradual decrease, while the hysteresis energy remained stable, showing 90% efficiency in the 50th cycle (Figure 5h,i). More impressively, the incorporation of HPC further enhanced the hydrogel’s crack resistance. This improvement can be attributed to the long HPC chains, which possess abundant active groups that both facilitate the formation of dense dynamic hydrogen bonds and promote chain entanglement within the cross-linked network (Figure 5j).^48^ As shown in Figure 5k, even in the presence of a notch, the hydrogel exhibited a fracture strength of 0.36 MPa and an elongation at break of 1328%, resulting in a high fracture energy of 66,250 J m^–2^. In contrast, the fracture energy of the hydrogel without HPC decreased markedly to 26,200 J m^–2^ (Figure S7).

### Self-Healing, Antifreezing, and Self-Adhesive Properties

Owing to the dynamic reversibility of hydrophobic associations and hydrogen bonding, the hydrogel exhibited excellent self-healing capability at room temperature. The hydrogel was cut in half and carefully reconnected in a 20 °C environment to facilitate the self-healing process. After 24 h of healing, the restored hydrogel could be stretched again, demonstrating its effective recovery (Figure 6a). Upon damage, the physically cross-linked points can reassociate, enabling efficient healing of the fractured regions and allowing the hydrogel to recover its original structure and properties within a relatively short period. With an increasing healing time, the tensile strength and strain at the break of the healed hydrogels gradually improved, as illustrated in Figure 6b. After 3 h of self-healing, the healing efficiency reached only 42%. However, when extended to 48 h, the hydrogel exhibited maximum tensile strength and breaking strain comparable to the original, with a healing efficiency of up to 93%. After five repetitions, the healing efficiency was maintained at 87% (Figure S8). As shown in Figure S9, with extended self-healing time, the transmittance gradually recovered, and after 48 h, the hydrogel became nearly transparent, with its transmittance approaching the original state. To further quantify the hydrogel self-healing process, rheological measurements were conducted. Across a wide range of strains at a constant angular frequency, the *G*′ values of the hydrogels remained significantly higher than their *G*″ values, indicating that the hydrogel networks were in a stable gel state instead of a sol state. After self-healing, the *G*′ values nearly return to their original state (Figure 6c). Based on the frequency range (ω = 0.1–100 rad s^–1^) sweep result, the hydrogel exhibited a similar performance in *G*′, indicating the successful reformation of the internal cross-linking network (Figure 6d). As shown in Figure 6e, repeated dynamic strain steps were performed, where γ increased from 0.1% to 500% to further evaluate the self-healing process of the hydrogel. Upon further increasing the applied strain, *G*′ and *G*″ decreased dramatically, and at a strain γ of 500%, *G*″ exceeded *G*′, indicating that the hydrogel transitions into a sol-state due to significant network disintegration. However, when the strain was reduced back to 1%, the hydrogels recovered to their initial values. This phenomenon can be attributed to the highly efficient reformation of the intrinsic network within the hydrogels.

**Figure 6:**
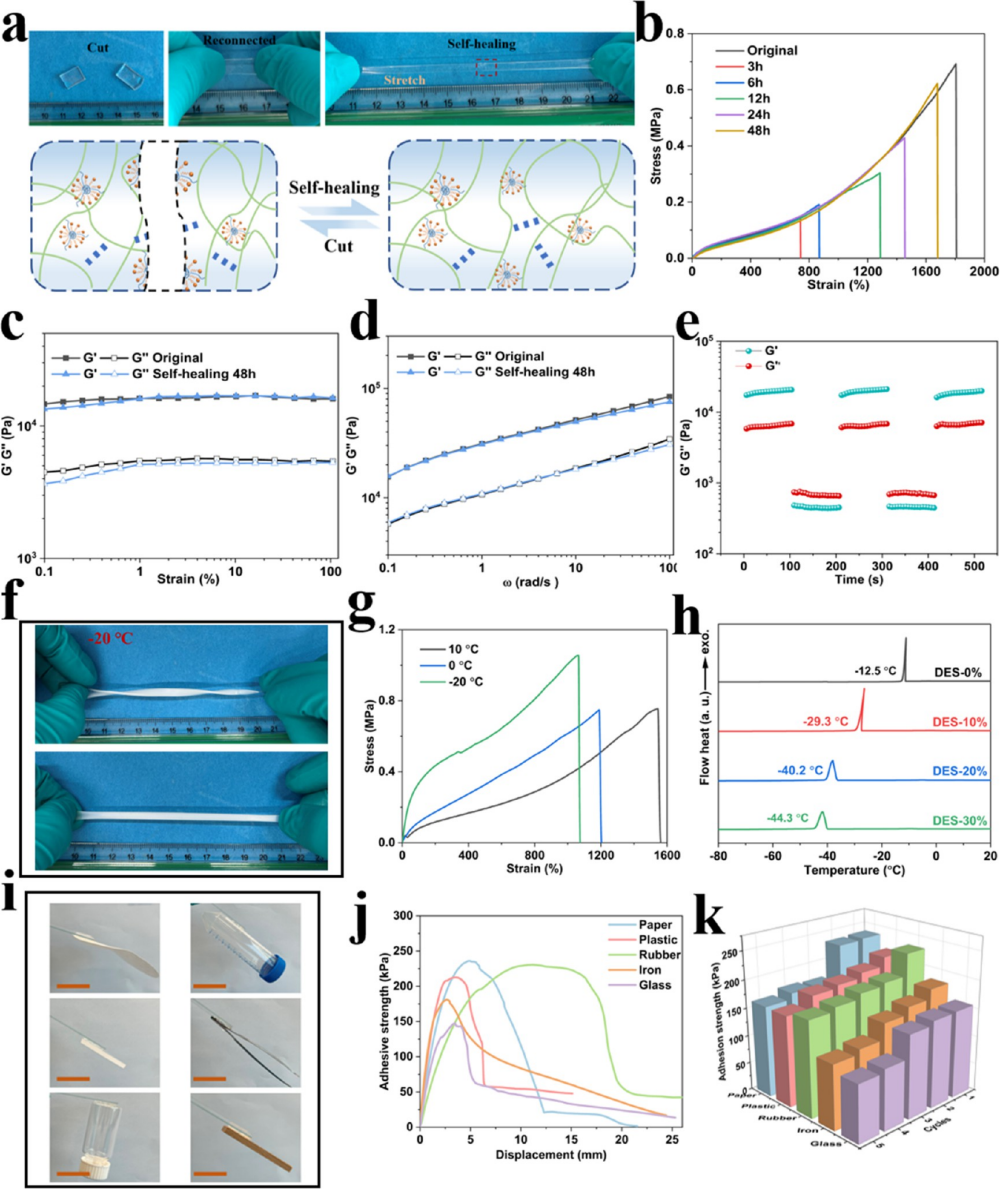
(a) Photographs showing the self-healing ability of the hydrogel and self-healing mechanism. (b) Stress–strain curves of the original and healed hydrogel after various healing times. (c) Variation of *G*′ and *G*″ with strain from 0.1 to 100% (ω = 1.0 Hz) for the original and healed hydrogels. (d) Variation of *G*′ and *G*″ with frequency from 0.1 to 100 rad s^–1^ for the original and healed hydrogel. (e) *G*′ and *G*″ dependence on time during continuous step strain measurements for hydrogels alternating between strain 1% and 500% (ω = 1.0 Hz). (f) Stretching and twisting hydrogels at −20 °C. (g) Stress–strain curves of the hydrogel at different temperatures. (h) DSC curves of hydrogels with different concentrations of DES. (i) Adhesion photographs of the hydrogel to various substrates. Scale bar: 2 cm. (j) Adhesion strength curves. (k) Five cycles adhesive strength curves.

The high water content makes hydrogels susceptible to freezing, leading to the formation of cracks and the loss of smart functionality at subzero temperatures. In this work, the incorporation of DES effectively mitigates water solidification. As shown in Figure 6f, the hydrogel containing DES retained its stretchability even after 24 h at −20 °C. Under tensile testing, the tensile strength increased to 1 MPa, while the elongation at break concurrently decreased to 1064% at −20 °C (Figure 6g). A series of DSC tests were conducted to investigate the freezing points of hydrogels with varying DES contents (Figure 6h). With the increase in DES content from 0 to 30 wt %, the freezing point of the hydrogel decreased from −12.5 °C to −44.3 °C. This phenomenon is attributed to the strong hydrogen bonding interactions between solketal in DES and water molecules, effectively inhibiting the formation of ice crystals.^49^

Hydrogels with strong adhesion can securely attach to substrates without the need for external adhesives, simplifying the installation in smart window applications. As illustrated in Figure 6i, the hydrogel exhibited outstanding self-adhesive properties across various substrates, including paper, plastic, rubber, iron, and glass, eliminating the need for auxiliary binders. The strong adhesion of the hydrogel is attributed to its side chains, which are rich in reactive groups such as amino and hydroxyl groups, enabling the formation of noncovalent interactions with reactive groups on the surface of various substrates.^11^ Lap shear tests further validated the adhesive strength of the eutectogel, achieving 236 kPa on paper, 213 kPa on plastic, 230 kPa on rubber, 180 kPa on iron, and 147 kPa on glass, respectively (Figure 6j). Furthermore, to confirm the hydrogel’s viability for repeated use, cyclic lap shear tests were conducted, revealing that it retained high adhesion strength across different substrates even after five cycles (Figure 6k).

### Energy-Saving Performance of the Hydrogel Smart Window

To further evaluate its energy-saving potential, laboratory simulation tests were conducted. A 2 mm thick hydrogel layer was sandwiched between two glass sheets, sealed, and installed in a model house, which was exposed to an infrared lamp as a heating source to simulate solar heating, with a temperature sensor placed inside to monitor room temperature variations (Figure 7a). The air-sandwiched and water-sandwiched windows with the same thickness were also installed as a control for comparison. Three types of windows were compared under identical test conditions, where they were heated by an infrared lamp for 600 s, followed by natural cooling after switching off the lamp. It was obvious that the interior temperature of the model house equipped with the air-sandwiched window was significantly increased from 19 to 45 °C due to the superior light transmittance of this window system. The interior temperature of the model house with the water-sandwiched window increased from 19 to 37.5 °C after 600 s of heating, which was 7.5 °C lower than that of the house with the air-sandwiched window (Figure 7b), due to the significantly higher specific heat capacity of water than that of air.^50^ In contrast, the temperature of the model house equipped with the hydrogel smart window rose more slowly to 28.5 °C after irradiation due to the hydrogel’s phase transition and remained lower than that of the house with an air-sandwiched window and a water-sandwiched window by 16.5 and 9 °C, respectively, until the infrared lamp was turned off. As the temperature increased, the hydrogel transitioned from a transparent to an opaque state, effectively blocking near-infrared light from entering the room. Figure 7c shows the temperature variations for the three types of windows under infrared lamp heating at different power levels. When the heating power increased from level 1 to 4, the hydrogel smart window exhibited a more stable thermal response, with the temperature rising by only 5 °C, compared to the air- and water-sandwiched windows, where the room temperature increased by 14 and 11.5 °C, respectively. The results confirm that the hydrogel smart window has excellent thermal insulation and room temperature regulation capabilities. To further assess the long-term stability of the hydrogel window, repeated heating and cooling cycles were conducted. Upon heating, the hydrogel underwent phase separation, becoming opaque, and the room temperature of the model house stabilized at 28.5 °C. Even after 10 cycles, the results remained consistent, demonstrating the hydrogel’s excellent repeatability and durability (Figure 7d).

**Figure 7:**
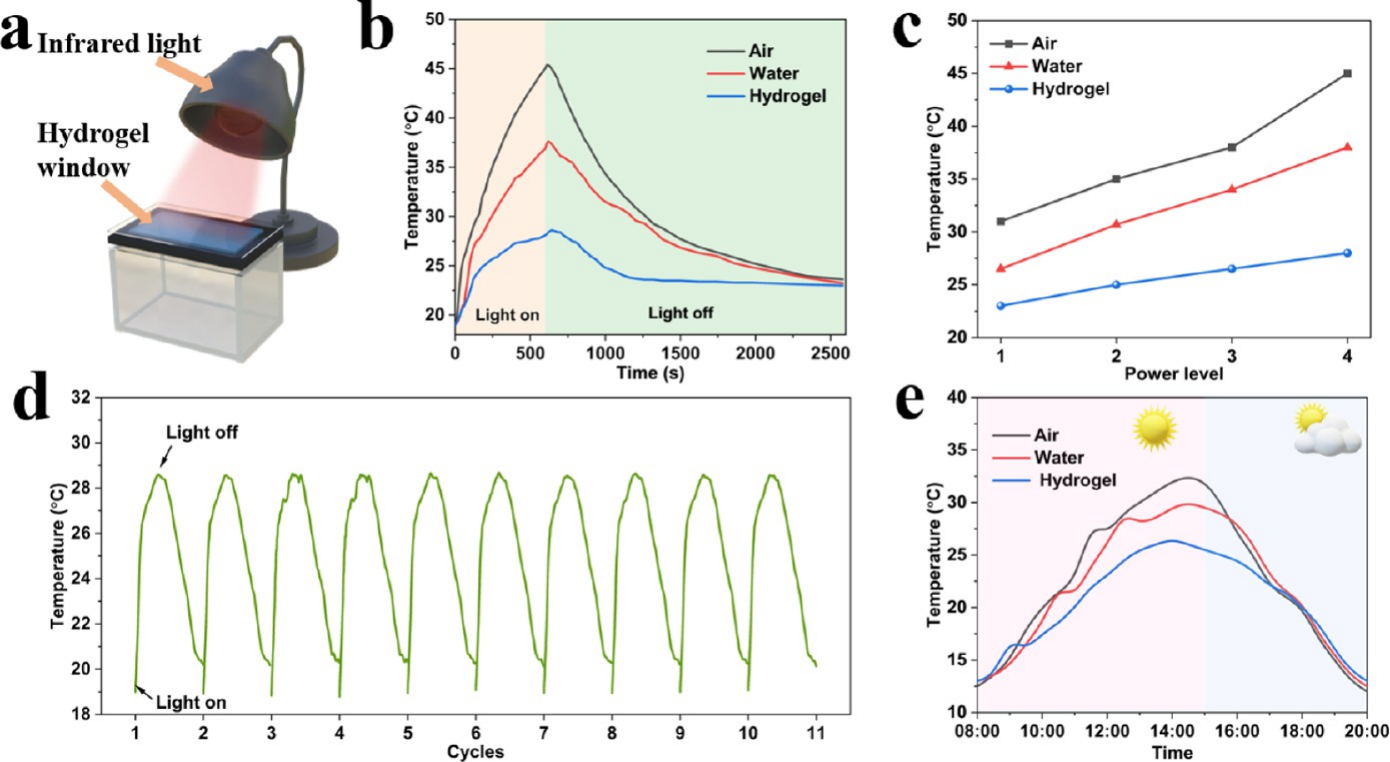
(a) Schematic illustrations of laboratory simulation tests. (b) The variation curves of simulated houses with three different window types under IR lamp irradiation. (c) Temperature variation curves of simulated houses with three different window types under different power levels. (d) Temperature changes in the model room after 10 cycles of IR lamp irradiation. (e) Temperature curves of simulated houses with three different window types exposed to solar radiation.

Additionally, the model house was placed outdoors on a sunny day in Groningen to monitor indoor temperature variations. Under sunlight exposure, the room temperatures of the air-sandwiched and water-sandwiched model houses continued to rise, reaching maximum values of 32 and 30 °C, respectively. In contrast, the room temperature of the hydrogel-equipped model house remained at 26 °C (Figure 7e) as the phase separation of the hydrogel effectively blocked sunlight and reduced heat accumulation. These results indicate that the hydrogel smart window can significantly reduce energy consumption by minimizing the need for air conditioning, effectively regulating indoor temperature and enhancing energy efficiency. Nevertheless, the hydrogel may not be suitable for colder regions where winter temperatures remain below *T*_c_. To address this, dual-responsive smart windows that couple thermochromic and electrochromic functionalities have attracted increasing interest. Leveraging the hydrogel’s intrinsic conductivity, external voltage can be applied to dynamically regulate light and heat transmission. Such synergy broadens climatic adaptability, enhances energy efficiency, and offers greater user control, making it a promising strategy for advanced building envelope technologies. We are actively working to address this challenge in our future research.

Furthermore, by leveraging the bidirectional optical response performance of the hydrogel, it holds great potential for applications in information security. As illustrated in Figure S10, the hydrogel and QR code were integrated into a sandwich-type structure to function as an information encryption device, offering a simple decryption method with high efficiency. When the temperature was either too low or too high, the QR code remained hidden and unintelligible. However, within a specific temperature range, the hydrogel became transparent, allowing the QR code to be clearly visible and easily scanned by smartphones or other devices. As the ULST and LCST can be finely tuned based on the above analysis, the temperature-responsive encryption labels can be precisely regulated, offering enhanced security and adaptability.

## Conclusions

In summary, a dual-temperature-responsive hydrogel was prepared in a DES/H_2_O mixed solvent by physically cross-linking an HEAA and AM copolymer with incorporated HPC, while CTAB was employed to stabilize LMA micelles. Physical cross-linking structures impart the hydrogel network with an ultrafast thermal response rate (30 s at 40 °C, 60 s at 6 °C) and excellent thermochromic performance. As a thermochromic smart window, the 2 mm thick hydrogel exhibits a high *T*_lum_ of 92%, and Δ*T*_sol_ reaches as high as 81.6% (ΔT_LCST_) and 83.6% (Δ*T*_c_), respectively. Upon decreasing the temperature, the hydrogel’s transmittance significantly decreases in the visible region, reaching a *T*_lum_ of 0.4%. The transparent temperature range of the hydrogel can be precisely controlled by tuning its composition, enabling thermochromic hydrogel smart windows to effectively regulate indoor temperatures and enhance energy efficiency across diverse climatic conditions. Moreover, owing to the dynamic reversibility of hydrophobic associations and hydrogen bonds, the hydrogel exhibits exceptional stress (0.68 MPa), high stretchability (1608%), and remarkable room-temperature self-healing performance (healing efficiency of 93% after 48 h), extending the service life of the smart hydrogel. The robust hydrogen bonding interactions between solketal molecules in the DES and water molecules significantly suppress the nucleation and growth of ice crystals, imparting superior antifreezing capabilities to the smart hydrogel window. The one-step polymerization avoids multistep processing and complicated preparation. The physically cross-linked hydrogel smart window could maintain structural integrity throughout phase transition, which is frequently compromised in covalently bonded analogs. This hydrogel-based smart window presents a significant advancement with the potential of simultaneously providing energy conservation and privacy protection, thereby opening new pathways for the development of next-generation bidirectional temperature-responsive materials.

## Experimental Section

### Materials

*N*-(2-Hydroxyethyl)­acrylamide (HEAA, 97%) and acrylamide (AM) were purchased from Merck. 1-Butyl-3-methylimidazolium chloride (BMIMCl, >98.0%), lauryl methacrylate (LMA, >98.0%), cetyltrimethylammonium bromide (CTAB, >98.0%), 2,2-dimethyl-1,3-dioxolane-4-methanol (solketal, >97.0%), 2-hydroxy-4′-(2-hydroxyethoxy)-2-methylpropiophenone (I2959, 99.0%), and hydroxypropyl cellulose (HPC, 3–6 mpas) were purchased from TCI.

### Preparation of DES

To prepare the DES, BMIMCl and solketal were mixed at a 1:2 molar ratio and stirred in a sealed flask at room temperature for 2 h until a clear, homogeneous liquid formed.

### Preparation of the Hydrogel

1 g of DES (1g, 20 wt % relative to water) was first mixed with 5 g of water, followed by the addition of HEAA and AM in a 1:1 molar ratio (total monomer mass: 3 g). Subsequently, 0.2 g of HPC and 0.25 g of LAM (both relative to water 4 and 5 wt %), along with 0.2 wt % of the photoinitiator I2959 (relative to the total monomer content), were incorporated to formulate the hydrogel precursor solution. The resulting precursor solution was poured into a custom-made reaction mold, which consisted of two rectangular glass plates and a hollow silicone rubber spacer (60 × 60 × 2 mm). Polymerization was carried out under UV irradiation (365 nm) for 10 min, forming the final hydrogel. Hydrogels with varying monomer ratios, different DES and LMA compositions, or altered HPC contents were prepared using the same procedure.

### Characterization

DLS measurements of LMA solutions were performed using a Mastersizer 3000 instrument (Malvern). Attenuated total reflection (ATR)–FTIR spectra were recorded over a wavenumber range of 500–4000 cm^–1^ using an IRTracer-100 (SHIMADZU), with data collected from 32 scans at a resolution of 4 cm^–1^. Rheological assessments were conducted by using a TA Instruments Discovery HR-2 rheometer equipped with an 8 mm parallel plate. The storage modulus (*G*′) and loss modulus (*G*″) of the hydrogels were evaluated across a strain range of 0.1–100% at a fixed angular frequency of 1 Hz. Frequency-dependent measurements of *G*′ and *G*″ were also performed within the range of 0.1–100 rad s^–1^ at room temperature under a constant strain of 1%. In addition, time-dependent *G*′ and *G*″ values were monitored by using a continuous step–strain test, where the strain amplitude was alternated between 1% and 500% at a constant frequency of 1.0 Hz. The hydrogels were placed at 6, 26, and 40 °C for 1 h and then immediately submerged in liquid nitrogen to preserve their microstructure at different temperature states, which was subsequently analyzed using a Nova NanoSEM 650. EDS was performed with the same instrument. UV–vis–NIR spectrometry (Agilent Cary 5000 Spectrophotometer) was employed to assess the transmittance over the wavelength range 200–2500 nm. The sample thickness was 2 mm. During testing, the samples were mounted onto a quartz glass sheet and secured in an instrument holder. Before measurement, the samples were heated to the designated temperature and maintained for 10 min to ensure thermal equilibrium. The integral luminous transmittance *T*_lum_ (380–780 nm), IR transmittance *T*_IR_ (780–2500 nm), solar transmittance *T*_sol_ (280–2500 nm), and corresponding transmittance modulations were calculated by eqs 1–3, respectively

1 $$T_{lum/IR/sol}=\frac{\int \varphi_{lum/IR/sol}\left(\lambda \right)T\left(\lambda \right)d\lambda }{\int \varphi_{lum/IR/sol}\left(\lambda \right)d\lambda }$$

2 ΔTlum/IR/sol,Tc=Tlum/IR/sol(at24C°)−Tlum/IR/sol(at6C°)

3 ΔTlum/IR/sol,LCST=Tlum/IR/sol(at24C°)−Tlum/IR/sol(at40C°)

where *T*(λ) denotes the recorded transmittance at a selected wavelength, φ_lum_ is the standard luminous efficiency function for the photopic vision of human eyes (wavelength coverage of 380–780 nm), and φ_IR/sol_ is the IR/solar irradiance spectrum for air mass 1.5.

The self-healing efficiency was calculated according to the formula *S*_0_/*S*_t_ × 100%, where *S*_0_ represents the original gel strain at break and *S*_t_ means the healed gel strain at break. To ensure reliability, each self-healing experiment was conducted on at least three independent samples, typically under ambient conditions, unless otherwise specified. DSC measurements were conducted using a TA Instruments DSC Q2000 instrument equipped with an RCS90 cooling system.

For the energy-saving performance test, a model house with dimensions of 20 × 15 × 10 cm^3^ was fabricated. The smart window, measuring 6 × 6 × 0.2 cm^3^, was assembled into the model by using a sandwich glass structure. A thermocouple thermometer (TA612C, TASI) was employed to monitor both the indoor temperature of the model and the ambient temperature. Double-glazed windows filled with air or water were used as comparative references. An infrared lamp (150 W) served as the simulated sunlight source for thermal control.

The mechanical properties of the composite gels were assessed by using a universal testing machine (SHIMADZU AGX-V) equipped with a 1 kN load cell. Dumbbell-shaped eutectogel specimens (20 mm × 8 mm × 2 mm) were subjected to tensile testing at a constant strain rate of 50 mm min^–1^. Young’s modulus was calculated from the slope of the stress–strain curve within the 5–15% strain range, while toughness was determined from the enclosed area of the stress–strain curve. Hysteresis energy was quantified based on the loading–unloading loop. The fracture toughness of the eutectogel was evaluated by using a pure shear test. Two sets of rectangular samples (width, *a*_0_ = 20 mm; thickness, *b*_0_ = 2 mm) were prepared, with a clamp-to-clamp distance of 5 mm. One set was unnotched, while the other was prenotched with an 8 mm slit introduced using a razor blade. The stress–strain response of the unnotched sample was recorded until fracture, and the area under the curve was defined as the work of deformation, denoted as *W*(*H*). The prenotched sample was then stretched until rapid crack propagation occurred, corresponding to the critical extension height *H*_c_. The fracture toughness (Γ) was calculated using the relation Γ = *W* (*H*_c_)/(*a*_0_ × *b*_0_). Hydrogel samples (20 × 15 × 2 mm^3^) were evaluated for lap-shear strength. Each sample was placed between two substrates, and a 1 kg weight was applied for 20 min to ensure full contact before testing. The tests were conducted at a loading rate of 20 mm min^–1^, and adhesion strength (τ_s_) was calculated as the maximum tensile force (*F*_max_) divided by the nominal contact area (τ_s_ = *F*_max_/*wl*), where *w* and *l* are the width and length of the contact area, respectively. Substrates were paper, iron, plastic, glass, and rubber.

## Supplementary Material







## Notes

Photographs of hydrogels noncovalently and covalently cross-linked; photographs of the precursor solution and hydrogel after polymerization containing CTAB-stabilized LMA micelles, without CTAB micelles, and with the addition of the chemical cross-linker MBA at 6 °C; hydrogel window with bidirectional response; mechanical properties, healing efficiency, and transmittance; and information encryption application (PDF)
Change in light transmittance when the hydrogel was exposed to 6 °C (MP4)
Change in light transmittance when the hydrogel was exposed to 40 °C (MP4)

## References

[1] Zhou Y., Dong X. X., Mi Y. Y., Fan F., Xu Q., Zhao H., Wang S. C., Long Y. (2020). Hydrogel smart windows. J. Mater. Chem. A.

[2] Zou X. T., Ji H. N., Zhao Y., Lu M. Y., Tao J. D., Tang P. H., Liu B., Yu X. T., Mao Y. L. (2021). Research
Progress of Photo-/Electro-Driven Thermochromic Smart Windows. Nanomaterials.

[3] Liu C. X., Yang L., Sun Y. X., Huang P., Yao Y., Tian Y., Zeng H. B. (2025). Hydrogel-Coated
Polydimethylsiloxane
with Reversible Transparency for Advanced Optical Switching. ACS Nano.

[4] He J. Q., Zhou Q., Ge Z. Q., Jiang S. F., Li J. H., Feng W., Yang H. Y. (2024). pH-Gated Switch of LCST-UCST Phase
Transition of Hydrogels. Adv. Funct. Mater..

[5] Xu G., Xia H., Chen P. Y., She W., Zhang H. N., Ma J., Ruan Q. S., Zhang W., Sun Z. M. (2022). Thermochromic Hydrogels
with Dynamic Solar Modulation and Regulatable Critical Response Temperature
for Energy-Saving Smart Windows. Adv. Funct.
Mater..

[6] Nan Y., Zhao C. Z., Beaudoin G., Zhu X. X. (2023). Synergistic Approaches
in the Design and Applications of UCST Polymers. Macromol. Rapid Commun..

[7] Gao X. Y., Cao Y., Song X. F., Zhang Z., Xiao C. S., He C. L., Chen X. S. (2013). pH- and
thermo-responsive poly­(-isopropylacrylamide-acrylic
acid derivative) copolymers and hydrogels with LCST dependent on pH
and alkyl side groups. J. Mater. Chem. B.

[8] Kawaguchi H., Fujimoto K., Mizuhara Y. (1992). Hydrogel Microspheres
Temperature-Dependent
Adsorption of Proteins on Poly-N-Isopropylacrylamide Hydrogel Microspheres. Colloid Polym. Sci..

[9] Chen G. Q., Wang K., Yang J. H., Huang J., Chen Z. F., Zheng J. X., Wang J. Q., Yang H. L., Li S. N., Miao Y. Y. (2023). Printable Thermochromic Hydrogel-Based Smart Window
for All-Weather Building Temperature Regulation in Diverse Climates. Adv. Mater..

[10] Wang K., Chen G. Q., Weng S., Hou L. X., Ye D. Z., Jiang X. C. (2023). Thermo-Responsive
Poly­(-isopropylacrylamide)/Hydroxypropylmethyl
Cellulose Hydrogel with High Luminous Transmittance and Solar Modulation
for Smart Windows. ACS Appl. Mater. Interfaces.

[11] Zhou B., Yuan W. Z. (2024). Tunable thermoresponsive
and stretchable hydrogel sensor
based on hydroxypropyl cellulose for human motion/health detection,
visual signal transmission and information encryption. Carbohydr. Polym..

[12] Feng Y. Q., Ma W. X., Li H. B., Yang M., Yu Y. Z., Liu S. M., Zeng X. L., Huang F., Yang Y. S., Li Z. H. (2023). Phase-Changing Polymer
Film for Smart Windows with Highly Adaptive
Solar Modulation. ACS Appl. Mater. Interfaces.

[13] Xie L., Wang X. C., Zou X. L., Bai Z. X., Liang S., Wei C., Zha S. Y., Zheng M. H., Zhou Y., Yue O. Y. (2023). Engineering Self-Adaptive Multi-Response Thermochromic Hydrogel for
Energy-Saving Smart Windows and Wearable Temperature-Sensing. Small.

[14] Song L. Z., Chen W. J., Huang J. R., Hu D. G., Ji X. X., Hua L., Lu Z. Q. (2025). Conductive
hydrogels with HPC additions for humidity
sensing and temperature response. Chem. Eng.
J..

[15] Shang J., Zhang Y. H., Zhang J. H., Zhang X. Y., An Q. (2024). Hydrogel-Based
Stimuli-Responsive Radiative and/or Evaporative Cooling Materials
for Carbon Neutrality. ACS Energy Lett..

[16] Wang K., Liu S., Yu J., Hong P., Wang W., Cai W., Huang J., Jiang X., Lai Y., Lin Z. (2025). Hofmeister
Effect-Enhanced, Nanoparticle-Shielded, Thermally Stable Hydrogels
for Anti-UV, Fast-Response, and All-Day-Modulated Smart Windows. Adv. Mater..

[17] Li G., Chen J. W., Yan Z. A., Wang S. C., Ke Y. J., Luo W., Ma H. R., Guan J. G., Long Y. (2023). Physical crosslinked
hydrogel-derived smart windows: anti-freezing and fast thermal responsive
performance. Mater. Horiz..

[18] Liu Y. H., Han Y. A., Huang Z. H., Qi P., Song A. X., Hao J. C. (2022). New focus of the cloud point/Krafft point of nonionic/cationic
surfactants as thermochromic materials for smart windows. Chem. Commun..

[19] Liu Y. H., Yan C. Y., Qi P., Song A. X., Hao J. C. (2023). Combining
Krafft Point and Volume Phase Transition Temperature Toward Regulation
of Solar Radiation and Privacy Protection. Adv.
Mater. Interfaces.

[20] Xu G., Lu Y. C., Zhou X. G. T., Moloto N., Liu J. C., Kure-Chu S. Z., Hihara T., Zhang W., Sun Z. M. (2024). Thermochromic
hydrogel-based energy efficient smart windows: fabrication, mechanisms,
and advancements. Mater. Horiz..

[21] Liu Y. H., Guo Y. Y., Zhang Z., Huang Z. H., Qi P., Cui J. W., Song A. X., Hao J. C. (2020). A new application
of Krafft point concept: an ultraviolet-shielded surfactant switchable
window. Chem. Commun..

[22] Caprioli M., Roppolo I., Chiappone A., Larush L., Pirri C. F., Magdassi S. (2021). 3D-printed self-healing hydrogels via Digital Light
Processing. Nat. Commun..

[23] Guo J., Wu S. S., Wang Y. L., Huang J. H., Xie H., Zhou S. B. (2022). A salt-triggered multifunctional smart window derived
from a dynamic polyampholyte hydrogel. Mater.
Horiz..

[24] Wang S. Y., Urban M. W. (2020). Self-healing polymers. Nat. Rev.
Mater..

[25] Yang L., Tan X., Wang Z., Zhang X. (2015). Supramolecular Polymers: Historical
Development, Preparation, Characterization, and Functions. Chem. Rev..

[26] Mohammad N. M., Zhang Y., Xu W. H., Aranke S. S., Carne D., Deng P. F., Du F. Y., Ruan X. L., Li T. (2024). Highly Tunable
Cellulosic Hydrogels with Dynamic Solar Modulation for Energy-Efficient
Windows. Small.

[27] Li B., Xu F. C., Guan T. T., Li Y., Sun J. Q. (2023). Self-Adhesive
Self-Healing Thermochromic Ionogels for Smart Windows with Excellent
Environmental and Mechanical Stability, Solar Modulation, and Antifogging
Capabilities. Adv. Mater..

[28] Li J. N., Lu X. G., Zhang Y., Wen X. X., Yao K. K., Cheng F., Wang D. C., Ke X. Q., Zeng H., Yang S. (2022). Dynamic Refractive Index-Matching for Adaptive Thermoresponsive Smart
Windows. Small.

[29] Wang W. Y., Wang K., Cheng Y., Wu C., Wu R. Z., Huang J. Y., Lai Y. K. (2024). Bidirectional Temperature-Responsive
Thermochromic Hydrogels With Adjustable Light Transmission Interval
for Smart Windows. Adv. Funct. Mater..

[30] Dai M. Y., Zhao J., Zhang Y. D., Li H. J., Zhang L. P., Liu Y., Ye Z. Y., Zhu S. M. (2022). Dual-Responsive Hydrogels with Three-Stage
Optical Modulation for Smart Windows. ACS Appl.
Mater. Interfaces.

[31] Guo R., Shen Y. C., Chen Y., Cheng C., Ye C. W., Tang S. C. (2024). KCA/NaSiO/PNIPAm hydrogel with highly robust and strong
solar modulation capability for thermochromic smart window. Chem. Eng. J..

[32] Gao W. C., Wang L. Y., Wei Q. Y., Wei Y. N., Ma H. N., Long L. X., Hou X., Zhao J., Yuan X. B. (2024). Pure Physical-Crosslinked
High-Strength Thermochromic Hydrogel for Smart Window and Energy Conservation. Adv. Funct. Mater..

[33] Lee H. S., Erwin A., Buxton M. L., Kim M., Stryutsky A. V., Shevchenko V. V., Sokolov A. P., Tsukruk V. V. (2021). Shape Persistent,
Highly Conductive Ionogels from Ionic Liquids Reinforced with Cellulose
Nanocrystal Network. Adv. Funct. Mater..

[34] Han S. W., Hu Y. K., Wei J., Li S. W., Yang P. P., Mi H. Y., Liu C. T., Shen C. Y. (2024). A Semi-Interpenetrating
Poly­(Ionic Liquid) Network-Driven Low Hysteresis and Transparent Hydrogel
as a Self-Powered Multifunctional Sensor. Adv.
Funct. Mater..

[35] Yao X., Zhang S. F., Qian L. W., Wei N., Nica V., Coseri S., Han F. (2022). Super Stretchable,
Self-Healing,
Adhesive Ionic Conductive Hydrogels Based on Tailor-Made Ionic Liquid
for High-Performance Strain Sensors. Adv. Funct.
Mater..

[36] Feng Y. Q., Wang S. C., Li Y. Q., Ma W. X., Zhang G., Yang M., Li H. B., Yang Y. S., Long Y. (2023). Entanglement
in Smart Hydrogels: Fast Response Time, Anti-Freezing and Anti-Drying. Adv. Funct. Mater..

[37] Jochum F. D., Theato P. (2013). Temperature- and light-responsive smart polymer materials. Chem. Soc. Rev..

[38] Fu M., Yuan Y. W., Liu X. B., Sun Z. X., Hu F. Q., Luo C., Yue K. A. (2025). A Thermosensitive Ionic Hydrogel for Thermotropic Smart
Windows With Integrated Thermoelectric Energy Harvesting Capability. Adv. Funct. Mater..

[39] Liu S., Li Y., Wang Y., Du Y. W., Yu K. M., Yip H. L., Jen A. K. Y., Huang B. L., Tso C. Y. (2024). Mask-inspired moisture-transmitting
and durable thermochromic perovskite smart windows. Nat. Commun..

[40] Yu Z. K., Ma Y. L., Mao L. H., Lian Y., Xiao Y. W., Chen Z. X., Zhang Y. H. (2024). Bidirectional
optical response hydrogel
with adjustable human comfort temperature for smart windows. Mater. Horiz..

[41] Lin C. J., Hur J., Chao C. Y. H., Liu G. Z., Yao S. H., Li W. H., Huang B. L. (2022). All-weather thermochromic windows for synchronous solar
and thermal radiation regulation. Sci. Adv..

[42] Wang Y. T., Fang X., Li S. H., An N., Pan H. Y., Sun J. Q. (2024). Polyelectrolyte complex-based thermochromic
hydrogels
containing carbonized polymer dots for smart windows with fast response,
excellent solar modulation ability, and high durability. Smartmat.

[43] Guan H. J., Lu Y. H., You Y. J., Gao S. X., Liu L., Wu G. F. (2024). Toughness and Thermoresponsive
Hydrogel for Sandwich Smart Window
with Adaptive Solar Modulation and Energy Saving. ACS Appl. Mater. Interfaces.

[44] Yu Z. K., Yang Y. Y., Shen C., Mao L. H., Cui C. C., Chen Z. X., Zhang Y. H. (2023). Thermochromic hydrogels with an adjustable
critical response temperature for temperature monitoring and smart
windows. J. Mater. Chem. C.

[45] Xie G. X., Li Y. F., Wu C. H., Cao M. R., Chen H. K., Xiong Y. J., Xu Y., Xie H. Q., Yu W. (2024). Dual response
multi-function smart window: An integrated system of thermochromic
hydrogel and thermoelectric power generation module for simultaneous
temperature regulation and power generation. Chem. Eng. J..

[46] Lei H., Dong L., Li Y., Zhang J. S., Chen H. Y., Wu J. H., Zhang Y., Fan Q. Y., Xue B., Qin M. (2020). Stretchable
hydrogels with low hysteresis and anti-fatigue
fracture based on polyprotein cross-linkers. Nat. Commun..

[47] Xu L. G., Huang Z. K., Deng Z. S., Du Z. K., Sun T. L., Guo Z. H., Yue K. (2021). A Transparent,
Highly Stretchable,
Solvent-Resistant, Recyclable Multifunctional Ionogel with Underwater
Self-Healing and Adhesion for Reliable Strain Sensors. Adv. Mater..

[48] Lu C. W., Wang X. Y., Shen Y., Xu S. J., Huang C. X., Wang C. P., Xie H. J., Wang J. F., Yong Q., Chu F. X. (2024). Skin-Like Transparent,
High Resilience, Low Hysteresis,
Fatigue-Resistant Cellulose-Based Eutectogel for Self-Powered E-Skin
and Human-Machine Interaction. Adv. Funct. Mater..

[49] Zhao Y. Q., Yang N., Chu X., Sun F. C., Ali M. U., Zhang Y., Yang B., Cai Y. L., Liu M. Y., Gasparini N. (2023). Wide-Humidity Range
Applicable, Anti-Freezing,
and Healable Zwitterionic Hydrogels for Ion-Leakage-Free Iontronic
Sensors. Adv. Mater..

[50] Zhou Y., Wang S. C., Peng J. Q., Tan Y. T., Li C. C., Boey F. Y. C., Long Y. (2020). Liquid Thermo-Responsive Smart Window
Derived from Hydrogel. Joule.

