# Supplementary material for: Thermoresponsive Physically Cross-Linked Hydrogels with Bidirectional Optical Response for Smart Windows Application
Source: ACS Appl Mater Interfaces. 2025 Sep 17;17(39):54967–80. doi: 10.1021/acsami.5c14919 (PMC12492321; doi:10.1021/acsami.5c14919)
Supplement: Supplementary file 1 [file am5c14919_si_001.pdf]

Supporting information

Thermoresponsive Physically Cross-Linked Hydrogels with Bidirectional Optical Response for Smart Windows Application

*Zeyu Zhang,[1] Aifang Yao,[2] Zao Cheng,[1] and Patrizio Raffa[1]**

1. Smart and Sustainable Polymeric Products, Engineering and Technology Institute Groningen (ENTEG), Faculty of Science and Engineering, University of Groningen, Nijenborgh 3, 9747 AG Groningen, The Netherlands

2. College of Biological Science and Engineering, Fuzhou University, Fuzhou 350108, China

* E-mail: p.raffa@rug.nl

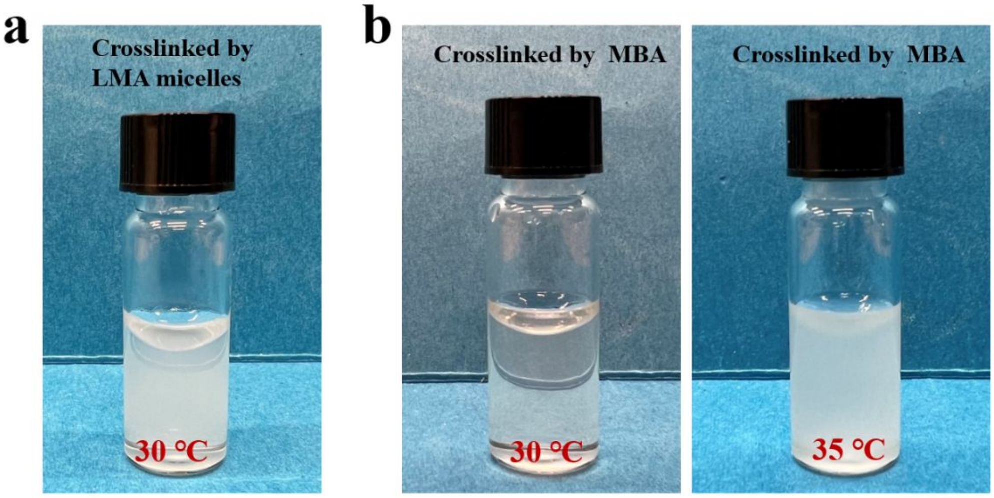

Figure S1. Photographs of hydrogels a) non-covalently crosslinked; b) covalently crosslinked

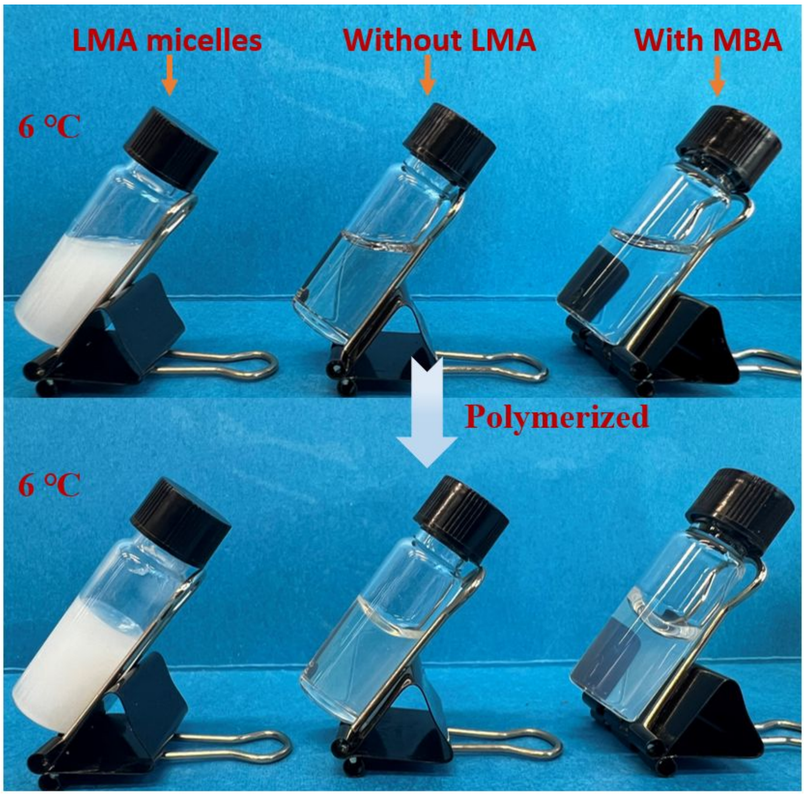

Figure S2. Photographs of precursor solution and hydrogel after polymerization containing CTAB-stabilized LMA micelles, without CTAB micelles, and with the addition of the chemical crosslinker MBA at 6 °C.

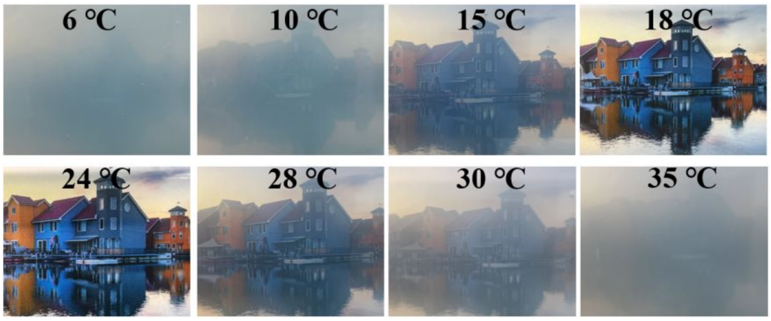

Figure S3. Photographs of hydrogel window with bidirectional response.

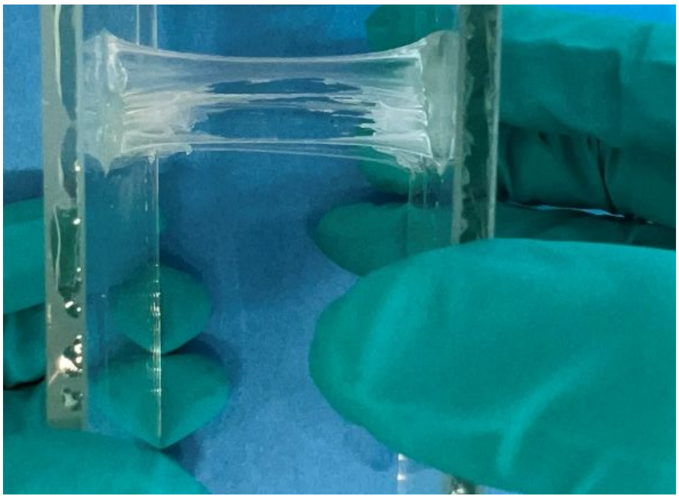

Figure S4. Photographs of $PHAL_0/DES_{20}/HPC_4$ hydrogel.

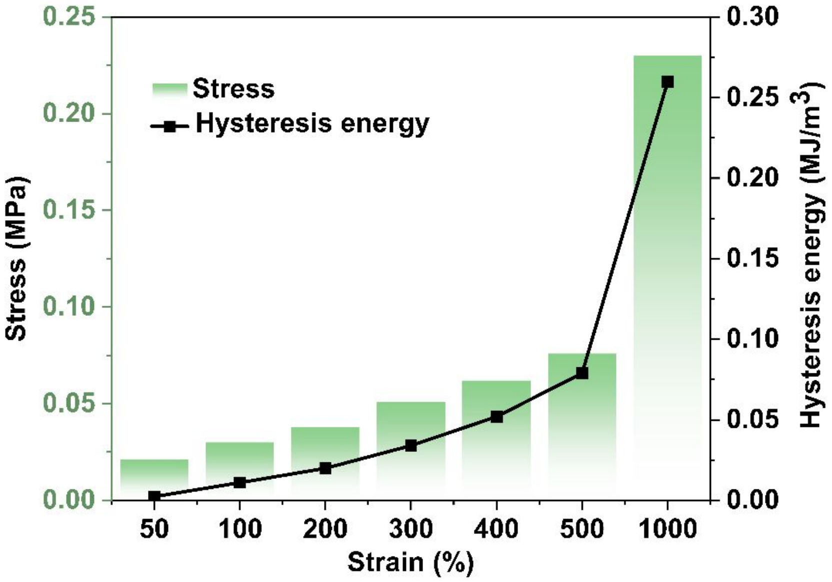

Figure S5. Stress and hysteresis energy of hydrogel with strain from 50 to 1000%.

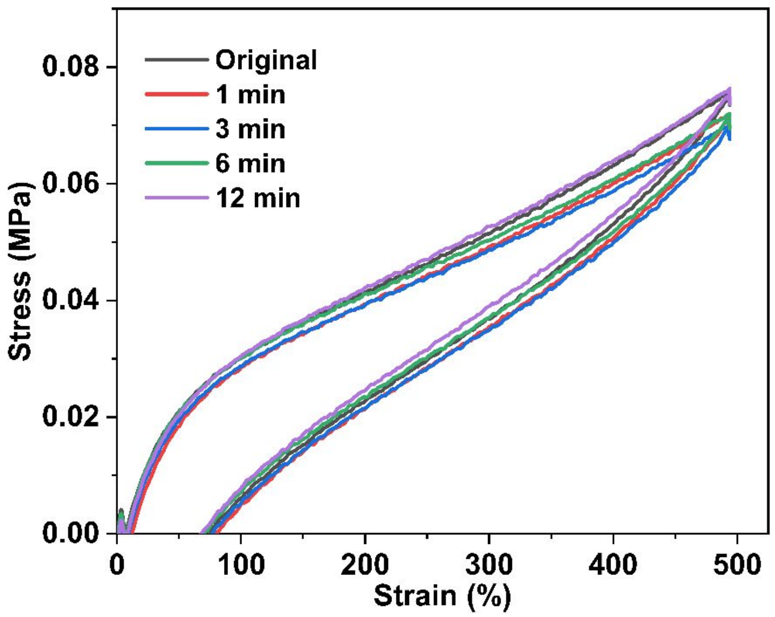

Figure S6. Stress-strain curves of hydrogel subjected to cyclic loadings in the stain of 500% with appropriate intervals.

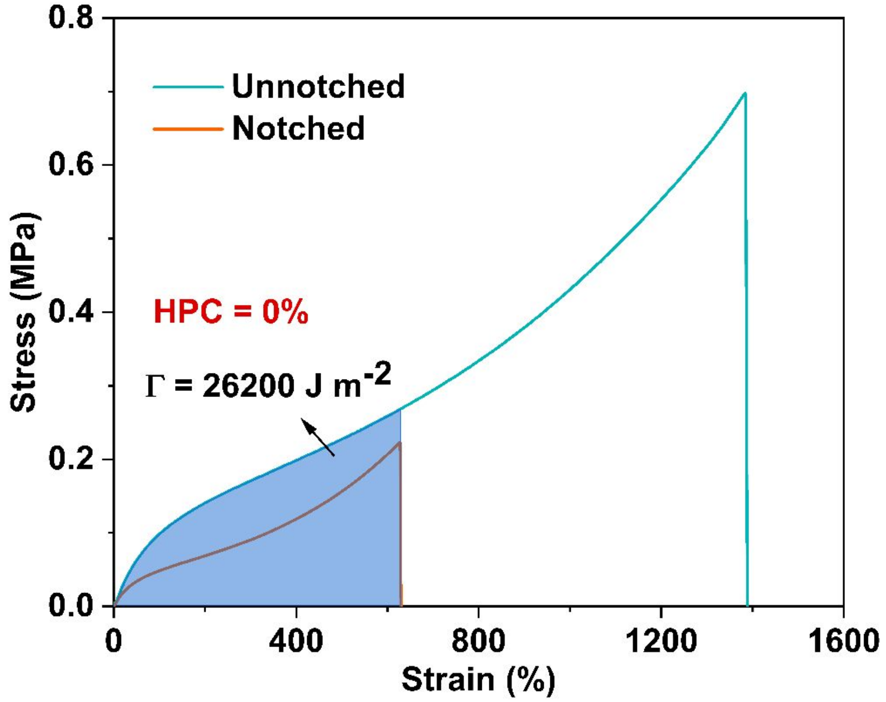

Figure S7. Tensile stress-strain curves of unnotched and notched hydrogels without HPC.

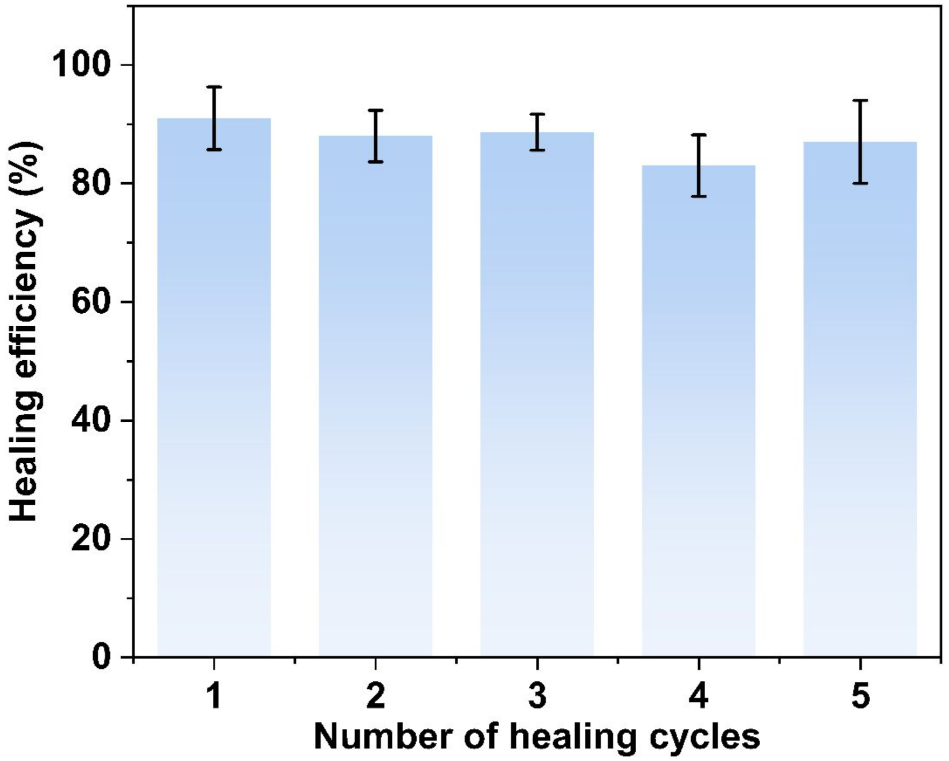

Figure S8. Healing efficiency of the $PHAL_5/DES_{20}/HPC_4$ hydrogel after different cutting and healing cycles.

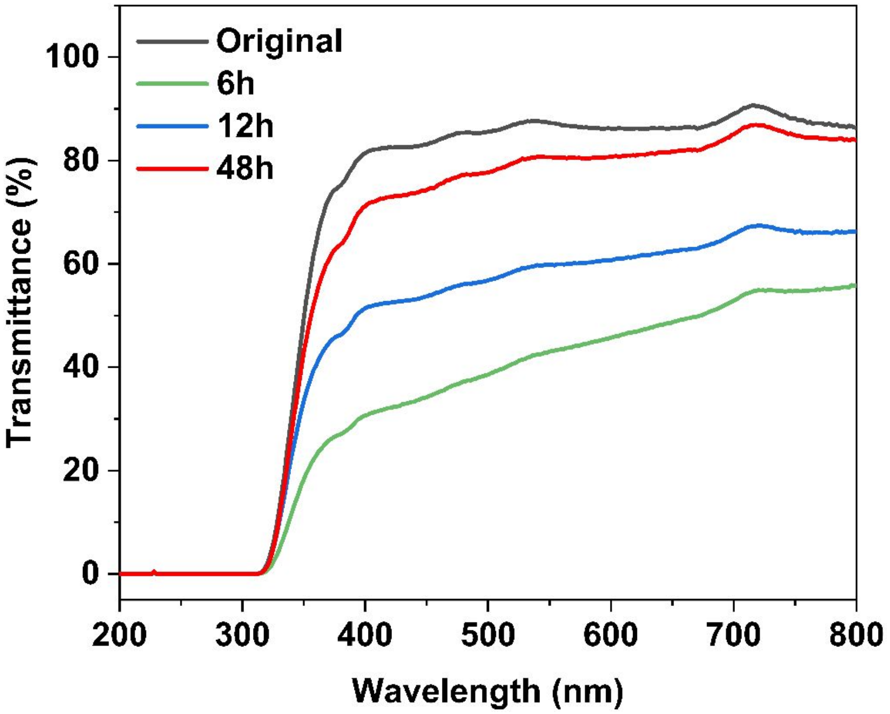

Figure S9. Transmittance spectra of the hydrogel after healing for different time.

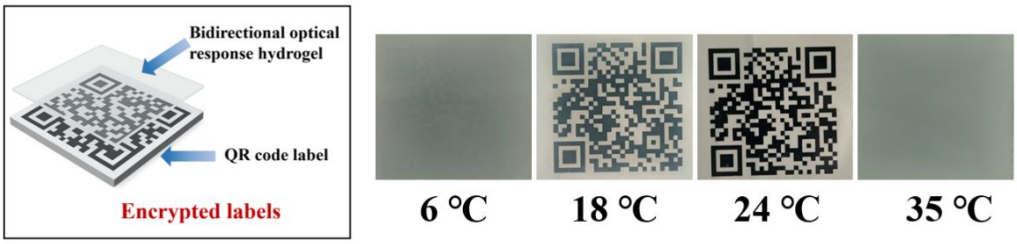

Figure S10. Schematic diagram of the multilayer tructured encrypted label based on hydrogel and photographs of the encrypted label of the multilayered structure based on hydrogel at different temperatures.
